# Do maternal demographics and prenatal history impact the efficacy of betamethasone therapy for threatened preterm labor?

**DOI:** 10.1186/s12884-021-03949-5

**Published:** 2021-06-24

**Authors:** Mary T. Kinney, Sara K. Quinney, Hayley K. Trussell, Larissa L. Silva, Sherrine A. Ibrahim, David M. Haas

**Affiliations:** 1 Division of Clinical Pharmacology, Indiana University School of Medicine, Indianapolis, USA; 2 Department of Obstetrics and Gynecology, Indiana University School of Medicine, 550 N. University Blvd, UH 2440, IN 46202 Indianapolis, USA

**Keywords:** Betamethasone, Antenatal corticosteroids, Preterm birth, Respiratory distress syndrome (RDS), Cesarean

## Abstract

**Background:**

Betamethasone (BMZ) is used to accelerate fetal lung maturation in women with threatened preterm birth, but its efficacy is variable and limited by the lack of patient individualization in its dosing and administration. To determine sources of variability and potential opportunities for individualization of therapy, the objective of this study was to evaluate maternal factors associated with development of neonatal respiratory distress syndrome (RDS) in a cohort of women who received betamethasone.

**Methods:**

This study prospectively enrolled women, gestational ages 23–34 weeks, who received betamethasone for threatened preterm birth. Maternal demographics, prenatal history, and neonatal outcomes were abstracted from hospital records. RDS was the primary outcome. Associations between RDS diagnosis and maternal demographics, prenatal history, and betamethasone dosing were evaluated in a case-control analysis and multivariable regression adjusted for gestational age at delivery. Secondary analyses limited the cohort to women who delivered within 1 or 2 weeks of betamethasone dosing.

**Results:**

Of 209 deliveries, 90 (43 %) resulted in neonatal RDS. Within the overall cohort and controlling for gestational age at birth, RDS was only associated with cesarean births compared to vaginal births (adjusted OR 1.17 [1.06–1.29]). Route of delivery was also the only significant factor related to RDS in the 83 neonates delivered within 7 days of BMZ dosing. However, among 101 deliveries within 14 days of betamethasone dosing and controlling for gestational age at birth, women who experienced preterm premature rupture of membranes (PPROM) had lower RDS rates than those without PPROM (57.9 % vs. 80.2 %, adjusted OR 0.81 [0.67–0.99]). Maternal age, BMI, race, and ethnicity were not associated with RDS in the regression models.

**Conclusions:**

Of maternal characteristics analyzed, only delivery by cesarean was associated with neonatal RDS after antenatal betamethasone use.

## Key points

1. Maternal factors do not predict BMZ efficacy to reduce RDS.
2. Babies born by cesarean delivery have higher rates of RDS.
3. Variability in BMZ efficacy should be further explored..

## Introduction

Preterm births, those occurring before the gestational age of 37 weeks, make up 12–13 % of all births in the United States and are the leading cause of neonatal morbidity and mortality [1]. Spontaneous preterm labor (40–45 %), preterm premature rupture of membranes (PPROM, 25–35 %), and indicated preterm deliveries for maternal or fetal health concerns (30–35 %) are the primary causes for preterm births [1]. Preterm neonates are at risk of fetal lung immaturity. With underdeveloped type II pneumocytes, the preterm neonate often has insufficient surfactant secretion, putting them at risk for respiratory distress syndrome (RDS). To accelerate fetal lung maturity and reduce the risk of RDS, antenatal corticosteroids (ACS) are given to women with gestational ages between 23 and 34 weeks who may deliver within 7 days. Betamethasone (BMZ) and dexamethasone are the most widely studied ACS used for this purpose. They rapidly cross the placenta to increase development of type 2 pneumocytes and subsequent secretion of surfactant [2–5].

ACS therapy is highly effective in reducing risk of RDS (by 34 %), but its efficacy is variable, with some infants benefitting and others still developing severe RDS [4]. Few changes have been made to the original schedule of ACS administration and dosing [2]. Previous studies suggested that individualization of BMZ dosing by maternal lean body weight reduces the variability in drug exposure, while other studies reported that maternal and umbilical cord BMZ concentrations were not affected by obesity [6, 7]. Other studies evaluated the effects of preeclampsia and plurality on neonatal outcome in the presence of BMZ administration without significant correlations found [6–8]. No personalized approach to ACS therapy currently exists.

The objective of this study was to examine if maternal characteristics predicted risk of RDS in a population of women receiving BMZ for threatened preterm birth. Our goal was to identify factors that may be utilized in explorations of a more individualized approach to BMZ therapy, in order to optimize newborn outcomes. Our hypothesis was that maternal characteristics would be independently associated with RDS.

## Methods

Women who were admitted to the Sidney & Lois Eskenazi Hospital and Indiana University Health Methodist Hospital in Indianapolis, IN with threatened preterm birth and who received BMZ for standard clinical care were recruited to the study between August 2016 and September 2019. Threatened preterm birth was defined as any woman who the provider believed was at-risk to deliver within the next 7 days such that the provider recommended ACS. Informed consent was obtained for all women enrolled and ethical approval was granted by the Indiana University Institutional Review Board. The two hospitals are urban county hospitals serving underserved patients and an academic medical tertiary care center, respectively. Participants had to be at least 18 years old and at least 23 weeks but less than 34 weeks of gestation with a live fetus. Exclusion criteria included known fetal anomaly, known placental abruption at the time of consent, multiple gestations, hepatic failure, renal failure, or inability to provide consent in English or Spanish. Women were recruited at either an initial or rescue course of BMZ as the primary aim of the study was a pharmacokinetic analysis. The original sample size was powered for the pharmacokinetic analysis. This is a secondary analysis of that parent study (NCT02793700).

The primary outcome of interest was neonatal RDS. RDS was diagnosed by the pediatricians following standard National Institute of Child Health and Human Development (NICHD) Neonatal Research Network criteria [9]. Standard maternal and delivery characteristics were collected by direct query or medical record review. Standard clinical care at the providers’ discretion was provided to the woman for anticipated/threatened preterm birth, and standard neonatal resuscitation and care practices were provided by the pediatric/neonatal services. As this was an observational study, no restrictions were placed on clinical provider care. Maternal age, body mass index (BMI), maternal ethnicity (Hispanic yes or no), race (African American, Caucasian, Other, Multiple, etc.), and other maternal and pregnancy characteristics were recorded for incorporation into the analysis.

The analysis was carried out using R in a case-control manner with the presence of RDS defining cases and infants who were not diagnosed with RDS serving as controls. A logistic regression model, controlling for gestational age at birth, was used to determine whether various maternal characteristics were associated with RDS and other neonatal outcomes. Subgroup analyses of women who delivered within 7 or 14 days of BMZ dosing were also performed. This time frame was chosen as it marks the patient’s eligibility to receive a rescue course of BMZ. Limiting this time frame allowed us to analyze preterm neonatal outcome and exclude women who proceeded to deliver at term. We also conducted a sensitivity analysis limited to women only receiving one course of BMZ.

## Results

**Fig. 1:**
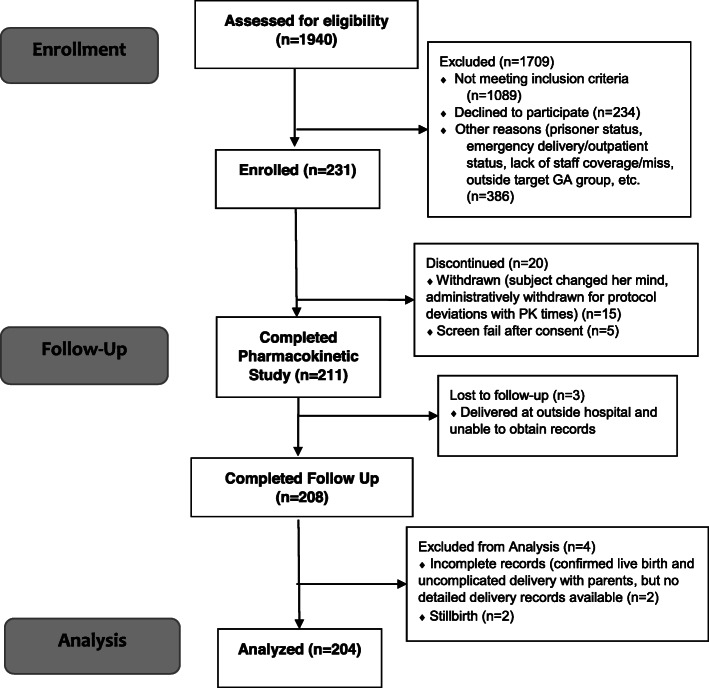
Flow diagram of individuals in the betamethasone study cohort

Of the 231 women initially consented for the study, 211 completed the study (Fig. 1). Three women delivered in out-of-state hospitals and we were unable to obtain delivery and infant outcomes as they were lost to contact. Two pregnancies resulted in stillbirths after recruitment: (1) 33 weeks gestational age at loss, 27 weeks at dosing, complicated by insufficient prenatal care and uncontrolled chronic hypertension; (2) 35 weeks gestational age, 33 weeks at dosing, complicated by gestational diabetes and morbid obesity. Two pregnancies were confirmed to have had live births, but we were unable to obtain hospital records with detailed newborn outcomes. 204 women completed the study with confirmed birth and neonatal RDS outcomes available. Of these women, 101 (48.3 %) deliveries resulted in neonates diagnosed with RDS. Table 1 displays the characteristics of women in the cohort whose newborns did and did not develop RDS. Mean gestational age (GA) at delivery for women whose babies developed RDS was 31.7 weeks vs. 36.9 weeks for women whose babies did not develop RDS (*p* < 0.001, Table 1). Neonates who developed RDS had higher rates of cesarean delivery (57/90, 63.3 %) than neonates who did not develop RDS (33/114, 28.9 %, *p* < 0.001).

**Table 1:** Demographic Characteristics of the cohort

Variable	Overall Cohort *n* = 209^a^	Neonates with RDS^b^ *n* = 90	Neonates without RDS *n* = 114	*p*-value^c^
Maternal Age	27.5 (5.9)	28.7 (6.4)	26.5 (5.1)	0.010
Maternal BMI^d^ at Dosing	32.4 (7.8)	32.0 (6.8)	32.7 (8.4)	0.488
Maternal Ethnicity				
Hispanic	37 (17.8%)	15 (16.7%)	22 (19.3%)	0.630
Non-Hispanic	171 (82.2%)	75 (83.3%)	92 (80.7%)	
Maternal Race				
Black or African American	90 (43.3%)	39 (43.3%)	48 (42.1%)	0.740
White or Caucasian	108 (51.9%)	48 (53.3%)	59 (51.8%)	
More than one Race/Other	8 (3.85%)	2 (2.2%)	6 (5.3%)	
Unknown/Not Reported	2 (0.96%)	1 (1.1%)	1 (0.9%)	
Maternal Alcohol Use	5 (2.4%)	3 (3.3%)	1 (0.9%)	0.209
Maternal Tobacco Use	56 (26.9%)	27 (30.0%)	28 (24.6%)	0.385
Maternal Illicit Drug Use	47 (22.6%)	21 (23.3%)	24 (21.0%)	0.696
Diabetes	28 (13.6%)	11 (12.2%)	16 (14.0%)	0.162
Preeclampsia	52 (25.2%)	31 (34.4%)	21 (18.4%)	0.007
Chronic Hypertension	44 (21.2%)	23 (25.6%)	21 (18.4%)	0.219
Premature Labor	72 (35.0%)	28 (31.1%)	43 (37.7%)	0.281
PPROM^e^	24 (11.5%)	14 (15.6%)	9 (7.9%)	0.086
GA^f^ at BMZ^g^ Dosing	30.8 (2.6)	30.0 (2.8)	31.5 (2.3)	<0.001
Days Between Last BMZ Dose and Delivery	25.6 (23.6)	10.9 (12.7)	37.0 (24.1)	<0.001
Total Doses of BMZ	2.3 (0.7)	2.4 (0.9)	2.2 (0.5)	<0.034
GA at Delivery	34.6 (3.6)	31.7 (2.7)	36.9 (2.2)	<0.001
Type of Delivery				
Vaginal	115 (55.3%)	33 (36.7%)	81 (71.1%)	<0.001
Cesarean Section	93 (44.7%)	57 (63.3%)	33 (28.9%)	
Male Fetal Sex	94 (45.4%)	40 (44.4%)	53 (46.5%)	0.771

^a^Five neonates did not have RDS outcomes recorded: two stillborn and three live births without traceable RDS outcome

^b^Respiratory distress syndrome (RDS)

^c^P-values are from univariable chi-squared analysis or Student’s t-test, comparing results between the group who developed RDS and the group that did not

^d^Body mass index (BMI) reported in kg/m^2^

^e^Preterm premature rupture of membranes (PPROM)

^f^Gestational age (GA)

^g^Betamethasone (BMZ)

After adjusting for GA at delivery, multivariable analysis showed that only cesarean delivery was significantly associated with RDS (adjusted OR 1.17 [1.06–1.29]). This relationship was present both for women who had a cesarean without or with labor (aOR 1.14 [1.03–1.27] and 1.25 [1.07–1.46], respectively). Maternal age, preeclampsia, gestational age at BMZ dosing, days between last BMZ dose and delivery, and total BMZ doses were significantly associated with RDS by univariable analysis in the overall cohort; however, after controlling for GA at delivery, these variables had no significant association with RDS so were therefore not included in the logistical regressions to evaluate other factors.

The subgroup analysis of newborns delivered to women within 14 days of their last BMZ dose included 1001 (48.3 %) women (Table 2). Within the subgroup, 57.9 % of neonates born to women with PPROM developed RDS vs. 80.2 % of neonates born to women without PPROM (adjusted OR 0.81 [0.67–0.99]). In this subgroup, women who had a cesarean with labor had higher odds of their infants developing RDS (aOR 1.42 [1.13–1.79]). No other factors were found to be associated with RDS in the subgroup. A second subgroup analysis evaluated infants born within 7 days of BMZ administration (Table 2). Findings were similar limiting it to babies born 2–7 days after initial BMZ dose (*n* = 27) and after sensitivity analysis limiting to women only receiving one course of BMZ (*n* = 171). After adjusting for gestational age at delivery, only cesarean delivery with labor was associated with a significantly higher risk of RDS (aOR 1.36[1.05–1.78]). In both the overall cohort and subcohorts, maternal demographics and prenatal factors such as maternal age, body mass index (BMI) at dosing, race, ethnicity, presence of diabetes, and prenatal substance use were not significantly associated with RDS after adjusting for GA at delivery (Table 1).

**Table 2:** Multivariable regression of factors associated with Respiratory Distress Syndrome development

	Adjusted Odds Ratios (CI)^**a**^				
	Overall Cohort	<14 Days^**b**^	<7 Days	2-7days	Only 1 course
N	209	101	83	27	171
Maternal Age	1.01 (1.00-1.02)	1.01 (0.99-1.02)	1.01 (1.00-1.02)	1.01 (0.98-1.04)	1.00 (0.99-1.01)
Maternal BMI^c^ at Dosing	1.00 (0.99-1.01)	1.00 (0.99-1.01)	1.00 (0.99-1.01)	1.00 (0.98-1.02)	1.00 (0.99-1.01)
Maternal Ethnicity, Hispanic	1.08 (0.95-1.22)	1.09 (0.89-1.32)	1.09 (0.88-1.35)	1.06 (0.71-1.57)	1.03 (0.91-1.17)
Maternal Race					
White or Caucasian	Reference	Reference	Reference	Reference	Reference
Black or African American	0.99 (0.90-1.09)	0.97 (0.81-1.17)	0.97 (0.81-1.17)	0.87 (0.62-1.21)	0.97 (0.88-1.07)
Other	0.89 (0.71-1.11)	0.76 (0.50-1.16)	0.76 (0.50-1.16)	0.49 (0.20-1.19)	0.90 (0.73-1.10)
Maternal Tobacco Use	1.03 (0.93-1.15)	1.04 (0.86-1.24)	1.05 (0.86-1.29)	1.23 (0.86-1.76)	1.03 (0.93-1.15)
Diabetes	0.92 (0.80-1.05)	1.03 (0.82-1.30)	1.12 (0.87-1.44)	1.08 (0.60-1.95)	0.92 (0.79-1.06)
Preeclampsia	1.07 (0.96-1.19)	1.08 (0.91-1.28)	1.04 (0.86-1.25)	1.05 (0.72-0.53)	1.06 (0.94-1.19)
Chronic Hypertension	1.06 (0.95-1.19)	1.09 (0.91-1.31)	1.08 (0.89-1.32)	1.22 (0.83-1.77)	1.11 (0.99-1.25)
PPROM^d^	0.91 (0.78-1.06)	0.81 (0.67-0.99)	0.85 (0.68-1.08)	0.82 (0.56-1.20)	0.89 (0.76-1.04)
Days Between Last BMZ^f^ Dose and Delivery	1.00 (1.00-1.00)	1.00 (0.98-1.02)	0.99 (0.94-1.04)	1.02 (0.91-1.16)	1.00 (1.00-1.00)
Total Doses of BMZ	1.06 (0.99-1.13)	1.01 (0.92-1.12)	1.00 (0.89-1.11)	1.14 (0.91-1.41)	0.83 (0.66-1.05)
Delivery Type					
Vaginal	Reference	Reference			
Cesarean without labor	1.14 (1.03-1.27)	1.13 (0.96-1.34)	1.14 (0.95-+1.38)	1.45 (1.07-1.98)	1.14 (1.03-1.27)
Cesarean with labor	1.25 (1.07-1.46)	1.42 (1.13-1.79)	1.36 (1.05-1.78)	2.08 (1.16-3.72)	1.24 (1.02-1.50)

^a^Odds ratios of maternal and prenatal factors by multivariable regression with adjustment for GA at delivery

^b^The subcohort refers to women who delivered within 14 days of receiving their last BMZ dose

^c^Body mass index (BMI) reported in kg/m^2^

^d^Preterm premature rupture of membranes (PPROM)

^e^Gestational age (GA)

^f^Betamethasone (BMZ)

## Discussion

In this study, we evaluated the impact of maternal characteristics and prenatal complications on the efficacy of BMZ therapy for threatened preterm labor to identify potential predictors of differences in neonatal RDS outcomes. We found that neonates delivered to women by cesarean have higher odds of developing RDS, which, in the overall cohort, is likely GA related. However, even when adjusting for GA at delivery, cesarean delivery remained associated with RDS, even when limiting to women delivered within 7 or 14 days of dosing. Previous studies have also shown an elevated risk of RDS in neonates born by cesarean. A recent meta-analysis by Li et al. found an association between cesarean section and RDS with a pooled odds ratio of 1.76 [1.48–2.09] [10]. Vaginal delivery may be protective against RDS due to mechanical forces that impact lung compliance and airway expansion after coming through the birth canal. Additionally, activation of sodium channels by endogenous catecholamines produced during labor further promote fluid clearance and may protect against RDS [11–14]. Very few studies, however, focus on neonatal RDS risk for cesarean births in the presence of BMZ therapy [10, 15]. Therefore, limited discussion exists regarding the role that BMZ plays in mediating the association between cesarean section and RDS.

In our subgroup of women who delivered within 14 days of BMZ administration, women whose admission diagnosis was PPROM had lower rates of RDS. This was not found in the Cochrane Review PPROM subgroup [16], however, our finding is similar those of studies by Berkowitz et al. and Sims et al. It is theorized that rupture of membranes promotes fetal cortisol production, which may further accelerate fetal lung maturation and decrease the risk of RDS [17–19]. This group made up a smaller proportion of the admission diagnoses for our cohort and thus this finding should be explored and replicated in other cohorts with more women admitted with PPROM.

Previous studies found that Black neonates were disproportionately represented in preterm births with low birth weight [20]. Other reports found that Black neonates have higher risk for perinatal death and more severe neonatal outcomes such as intraventricular hemorrhage, sepsis, and retinopathy of prematurity, though risk of RDS was not increased [21, 22]. This is congruent with our findings. Our study looked at women diagnosed with threatened preterm delivery for which they received BMZ but may or may not have delivered preterm. This broadens our population from those studies which found racial and ethnic disparities associated with poor premature neonatal outcomes and may contribute to the difference in findings.

Advanced maternal age has previously been associated as a risk factor for RDS [23]. Similar to Condò et al., we did not find an association between maternal age and RDS [15].

Neonates born to women with diabetes during pregnancy are thought to have a higher risk of RDS. Most of the data supporting this theory, however, originate from studies outside the setting of preterm birth with routine ACS use [24–26]. When focusing specifically on preterm deliveries, Bental et al. found no increase in the risk of RDS in the presence of maternal diabetes. Although not all women in their study received ACS, mothers with diabetes were more likely to receive and complete a full ACS dose [27]. While ACS may benefit the babies of women with diabetes, we did not have data on the adequacy of glucose control during the dosing or at the time of delivery. Thus, we are unable to comment on adverse events for a woman’s blood sugars. These factors should be investigated in future projects.

Finally, we did not find a significant association between maternal BMI and RDS. Previous studies have similarly found no significant association between pre-pregnancy BMI and RDS [28, 29]. Our study expands upon these findings because we measured BMI at the time of BMZ dosing to account for body weight changes during pregnancy. Gyamfi et al. quantified the lack of association by measuring the concentration of BMZ in maternal serum and cord blood at delivery and found no association between either of the BMZ concentrations with BMI [7]. Alternatively, a recent study looked at adipose tissue content rather than BMI and found that maternal mean body fat mass, fat ratio, truncal fat mass, and truncal fat ratio were significantly higher in mothers whose babies developed RDS [30]. We plan to incorporate these measures in future work.

As an observational case-control study, our study was limited in that clinical care was not dictated by the protocol and thus timing of BMZ may have been altered by providers due to clinical situations. All women did receive standard 12 mg dosing of BMZ, however. We were also limited in that we did not capture other characteristics of the labor progress. This will be explored in future studies. It is also possible that our sample was too small to detect differences in some of the maternal characteristics for the outcome groups.

In conclusion, we found that cesarean delivery was independently associated with increased odds of neonatal RDS in women receiving BMZ therapy for threatened preterm birth. While we did not identify maternal factors predictive of RDS, we continue to explore other factors, such as pharmacokinetic parameters or other biochemical biomarkers, which could lead to an improved predictive model of RDS and other newborn outcomes. In those ways, we can evaluate and develop a more individualized therapeutic strategy for ACS in threatened preterm birth.

## Data Availability

Not applicable.
